# Internationally educated nurses in Canada: predictors of workforce integration

**DOI:** 10.1186/s12960-017-0201-8

**Published:** 2017-04-04

**Authors:** Christine L. Covell, Marie-Douce Primeau, Kelley Kilpatrick, Isabelle St-Pierre

**Affiliations:** 1 Faculty of Nursing, University of Alberta, 5-301, ECHA, 11405-87 Avenue, Edmonton, Alberta T6G 1C9 Canada; 2 SETYM International, 80, Ste-Catherine Ouest, Montréal Québec, H2X 3P4 Canada; 3 Faculty of Nursing, Université de Montréal, Hôpital Maisonneuve-Rosemont, CSA-RC-Aile bleue-Room F121, 5415 boul. l’Assomption, Montréal, Quebec H1T 2M4 Canada; 4 Department of Nursing, Université du Québec en Outaouais [UQO], 283 Alexandre-Taché, Room C-1601, Gatineau, Québec J8X 3X7 Canada

**Keywords:** Internationally educated nurses, Cross-sectional survey, Immigration, Policy, Professional recertification, Employment, Workforce integration, Human capital, Canada

## Abstract

**Background:**

Global trends in migration accompanied with recent changes to the immigrant selection process may have influenced the demographic and human capital characteristics of internationally educated nurses (IENs) in Canada and in turn the assistance required to facilitate their workforce integration. This study aimed to describe the demographic and human capital profile of IENs in Canada, to explore recent changes to the profile, and to identify predictors of IENs’ workforce integration.

**Methods:**

A cross-sectional, descriptive, correlational survey design was used. Eligible IENs were immigrants, registered and employed as regulated nurses in Canada. Data were collected in 2014 via online and paper questionnaires. Descriptive statistics were used to examine the data by year of immigration. Logistic regression modeling was employed to identify predictors of IENs’ workforce integration measured as passing the licensure exam to acquire professional recertification and securing employment.

**Results:**

The sample consisted of 2280 IENs, representative of all Canadian provincial jurisdictions. Since changes to the immigrant selection process in 2002, the IEN population in Canada has become more racially diverse with greater numbers emigrating from developing countries. Recent arrivals (after 2002) had high levels of human capital (knowledge, professional experience, language proficiency). Some, but not all, benefited from the formal and informal assistance available to facilitate their workforce integration. Professional experience and help studying significantly predicted if IENs passed the licensure exam on their first attempt. Bridging program participation and assistance from social networks in Canada were significant predictors if IENs had difficulty securing employment.

**Conclusions:**

Nurses will continue to migrate from a wide variety of countries throughout the world that have dissimilar nursing education and health systems. Thus, IENs are not a homogenous group, and a “one size fits all” model may not be effective for facilitating their professional recertification and employment in the destination country. Canada, as well as other countries, could consider using a case management approach to develop and tailor education and forms of assistance to meet the individual needs of IENs. Using technology to reach IENs who have not yet immigrated or have settled outside of urban centers are other potential strategies that may facilitate their timely entrance into the destination countries’ nursing workforce.

## Background

In this paper, we present the profile of internationally educated nurses (IENs) in Canada, explore recent changes to the profile, and identify key factors that influence their workforce integration. Understanding how to facilitate the workforce integration of IENs has implications for health workforce planning and immigration policy, internationally. Canada offers an excellent case to examine IEN workforce integration due to the professional and financial commitments made to this group of immigrant health providers. The evidence generated from this study can be used internationally to develop policies and programs to support the workforce integration of IENs and other internationally educated health professionals.

Considerable international importance has been given to IENs as a potential solution for addressing health workforce shortages in developed countries [1–3]. For some time, Canada, the United States, the United Kingdom, and Australia have been top destination countries for IENs [4]. In recent years, these nations as well as other developed countries in Europe have experienced dramatic increases in the numbers of IENs [5–7]. Most of the documented “push” and “pull” factors that influence IENs’ decisions to migrate are fuelled by the assumption that nurses possess a tangible and transferable skill set that is suitable for gaining employment, globally [1].

Migration can have significant influence on the destination and source countries and IENs and their families. Destination countries benefit from gaining experienced nurses needed to “rapidly” fill gaps in their health workforce while source countries lose valuable health human resources often exacerbating existing critical shortages [8]. IENs can encounter significant obstacles when attempting to integrate into the destination country’s nursing workforce, which causes some to abandon their profession and suffer financial hardships [9]. These circumstances led to international cries for the ethical recruitment and treatment of health professionals [10–12].

### Canadian context

#### Internationally educated nurses in Canada

Canada has an active program for permanent immigration and a long history of welcoming IENs. In the last decade, there was a dramatic increase in the number of IENs, many from developing countries, who migrated to Canada [13]. Some have independently migrated through formal immigration channels; others were recruited to work in healthcare environments through private or provincial initiatives [14]. Many IENs have become permanent Canadian residents [15].

Data from the Canadian Institute for Health Information (CIHI) indicates the Philippines and the United Kingdom are primary source countries for the 25 656 (7.3%) regulated nurses who are internationally educated [13]. However, estimating the exact number of IENs in Canada remains difficult. This is because not all immigrants provide information about their occupation and not all IENs decide to pursue professional recertification. An absence of data systems to collect information about the immigration and integration of IENs further limits our abilities to identify the IENs who are not registered with a regulatory body, to describe the IEN population in Canada, or to explain why some IENs are unable to practice their profession [16].

#### Canadian immigration policy

Since 2002, the Canadian immigration policy and the selection of immigrants has been guided by the *Immigration and Refugee Protection Act* (IRPA) [17]. The major goals of the immigration policy, as outlined by the IRPA, are as follows: reuniting families, contributing to economic development, and protecting refugees [18]. Accordingly, a human capital approach was applied to the selection of immigrants that awards higher points for applicants’ education level, language abilities, and professional experience. Other highly weighted characteristics include the applicants’ potential to adapt to Canadian society, including age, spouses’ education, previous Canadian work experience, and relations in Canada [19]. The IRPA supports the permanent immigration of individuals with higher levels of human capital (characteristics of professional knowledge, skills, and experience) and social capital (forms of assistance: social and professional networks) in Canada without placing limits on their race, ethnicity, or where they were born or educated [17].

#### Internationally Educated Health Professionals Initiative

Growing concerns arose over the numerous internationally educated health professionals, including nurses, who were struggling to become recertified or were unable to practice their profession in Canada [20]. As a result, several million dollars, both public and private, were devoted to facilitate the professional integration of internationally health professionals. One such example is the initiative funded by the Canadian government, the Internationally Educated Health Professionals (IEHP) Initiative. The IEHP Initiative provides financial support for both pan-Canadian and provincial/territorial initiatives to develop consistent approaches for integrating IEHPs into the Canadian health workforce. The IEHP Initiative led to the creation of many innovative programs and supports to help IEHPs traverse the professional recertification process and job search in Canada [21]. However, little research has been conducted on the appropriateness and effectiveness of these initiatives in facilitating IENs’ workforce integration.

#### Workforce integration of IENs in Canada

The workforce integration of IENs in Canada consists of two significant milestones: (1) achieving professional recertification as a regulated nurse, accomplished by achieving success on a licensure exam, and (2) securing employment as a regulated nurse. Thus, for this study, we define IEN workforce integration as IENs acquiring professional recertification and securing employment as regulated nurses in Canada. In the following section, we review the literature about the facilitators of professional recertification and employment of IENs in Canada.

##### Facilitators of professional recertification

Prior to 2014,^1^ professional recertification in Canada involved nurses having their professional credentials formally verified and assessed through the appropriate provincial or territorial nursing regulatory body [16]. In all jurisdictions, to become eligible to take a licensure exam, nurses must provide evidence of identity, educational equivalence, previous registration as a nurse in another country (if applicable), evidence of suitability and ability to practice nursing safely, and demonstrate language competency (English and/or French) [22].^2^ A baccalaureate in nursing is required for entry to practice as a registered nurse in all jurisdictions of Canada, except Quebec [23].

IENs can take much longer than the 1 year it takes their Canadian-educated counterparts to pass the licensure exam [22]. Perfecting their language skills, learning the occupational-specific vocabulary used in the workplace, and understanding the scope of nursing practice in Canada have been identified as facilitators of IENs passing the licensure exam [24]. IENs report using a variety of strategies to gain the knowledge required to achieve success on the licensure exam, including reading nursing journals and exam review books, perfecting their language skills, and acquiring mentorship or tutoring from friends or colleagues in Canada [25]. IENs also report that participating in IEN bridging programs helped them pass the licensure exam [26]. Although bridging program curricula differ, in general they are designed to assist IENs with workforce integration by offering courses to update their professional competencies to meet Canadian standards, to learn occupational-specific language, and to prepare for the licensure exams [27]. Many bridging programs include courses about applying and interviewing for nursing positions [28].

##### Facilitators of employment

Once recertified, IENs must search for employment [22]. Some IENs pre-arrange their employment before migrating, but most wait until after they enter Canada to begin their job search [29]. Many IENs assume because they were admitted to Canada, a country where nurses are “needed,” finding employment in their chosen area or locality will be relatively easy [20]. Yet, depending on the economy and the job market, securing employment in their chosen setting or clinical area may be challenging for some IENs [30]. Becoming acquainted with the different sectors and settings within the Canadian healthcare system and learning how to apply for jobs and to complete the interview process [31] are strategies IENs can use to accelerate their employment. Oftentimes, IENs rely on their social network of friends and family in Canada to help them navigate the job search and hiring process [32].

IENs seem more likely to be hired if they participate in bridging programs, have professional experience in Canada or an equivalent healthcare setting, have strong language skills, and are willing to work in hard-to-fill sectors of the healthcare system such as long-term care or regions of the country with vacancies such as rural or remote areas [33]. Employers report that to be hired, IENs must have the necessary knowledge, nursing competencies, professional experience, and language skills to practice safely [16, 23].

The overall purpose of this study was twofold: (1) to describe a demographic and human capital profile of IENs in Canada and to explore recent changes in the profile and (2) to identify the key human capital characteristics and types of assistance that predict IENs’ professional recertification and employment as regulated nurses.

## Methods

### Design and setting

A cross-sectional, survey design was used to collect information about the demographic and human capital characteristics of the IEN population in Canada and the types of assistance that facilitated their professional recertification and employment. Data were collected from IENs in 10 provinces and 2 territories of Canada. Collecting data in the Yukon was not feasible due to the very small number of IENs employed in the territory [34].

### Participants

Regulated nurses were eligible to participate in the study if they were immigrants and obtained their basic nursing education in a country other than Canada. Only IENs who had permanent licenses to practice nursing as registered nurses (RNs), licensed practical nurses (LPNs), or registered psychiatric nurses (RPNs) and were employed as regulated nurses in a Canadian jurisdiction at the time of the survey were targeted.

### Questionnaire

The questionnaire developed for this study drew on the work of Primeau [35], who investigated the influence of participation in bridging and employer orientation programs on IENs’ successful completion of a 90-day employer probation period and 1-year retention with healthcare organizations in Quebec. We used the research evidence plus findings generated from a scoping review of the literature about the integration experiences of IENs in Canada to identify the forms of assistance available to support IENs’ professional recertification and employment [16]. The questions were developed to reflect the greater Canadian context (inclusion of all regulatory nursing professions and jurisdictions) and to more fully explore the human capital characteristics (educational preparation, language proficiency, and professional experience) and forms of assistance (formal assistance: bridging program participation and informal assistance: help from colleagues, family, friends) that may influence IENs’ integration, career advancement, and retention.

The questionnaire had three sections. The first section inquired about IENs’ integration experiences including workforce integration (achieving professional recertification and securing employment) and workplace integration (becoming a member of a workgroup within an organization). The second section had questions about IENs’ career advancement opportunities (career goal achievement and satisfaction) and retention intentions (retaining IENs in the nursing profession in Canada). The survey ended with questions about participants’ demographic and human capital characteristics. The questionnaire was composed of fixed-response items, Likert-type scales, and open-ended (fill-in) questions. It had 60 items and took approximately 30 min to complete.

### Data collection procedures

The questionnaire was subjected to pilot tests for content and face validity and test-retest reliability using a panel of experts recruited for their knowledge about Canadian IENs [36]. Their feedback was used to refine the questions prior to their use in this study. In order to provide the questionnaire in the participants’ preferred language, the items were translated into the French language by following the process for cultural adaptation and translation for research tools [37]. The online versions of the questionnaire were pilot-tested for functionality prior to their use in the study.

IENs were identified through the nursing regulatory bodies in the 10 provinces and 2 territories of Canada. The recruitment strategies varied with the regulatory bodies’ policies for research notification: 19 regulatory bodies assisted with identifying IENs, of which 11 contacted the IENs on our behalf, 5 provided the researchers with the IENs’ contact information, and 3 posted the study invitation on their websites or in their newsletters. IENs who agreed to be contacted for research purposes were contacted by electronic or postal mail.

Data were collected between February and October 2014, following the Tailored Design Method for Survey implementation [38]. A total of 13 748 IENs were mailed an invitation letter to participate in the study and the questionnaire. Three reminders were used to increase the response rate. Participants were able to complete the questionnaire in English or French, anonymously online or by postal mail. Identifying information provided by the participants was removed prior to analysis and stored separately to maintain confidentiality and to control for potential response bias.

CIHI reports 25 656 IENs are registered with a regulatory body in Canada [34]. We estimated that 45–50% would have given permission to be contacted for research purposes, of which 30% (*N* = 3848) would respond to our invitation and return the completed questionnaire [38]. A sample of 3848 participants would provide sufficient power to describe the IENs’ profile, to compare the profile of IENs before and after 2002 (anticipating a small difference between groups, *p* = .01, *β* = .80), and to examine the influence of multiple (8) factors on workforce integration [39].

### Variables

The variables used in this study reflect the demographic and human capital characteristics of IENs and the forms of assistance available to support their workforce integration. The participants reported a wide range of responses for some of the variables. The responses for these variables were collapsed into meaningful categories in order to balance the number of participants across categories, minimize the potential problem of unequal variance, and facilitate the interpretation of the estimated parameters.

#### Demographic variables

The demographic variables for this study included gender, age, visible minority, country of education, year of immigration, Canadian regulatory status, and jurisdiction of registration. Gender was measured as a categorical variable (1 = male, 2 = female). Age was reported in years. Participants were asked to identify if they were members of a visible minority racial group by using the categories included in the Canadian Census [40]: Black, Chinese, South Asian, Filipino, Arab, Korean, Southeast Asian, Japanese, Latin American, West Asian, and Mixed Race. Nonvisible minority was categorized as White or Caucasian. Participants’ responses were grouped into visible minority (1) or not visible minority (0).

Country of education was assessed with an open-ended question where the participants were asked to write in their country of basic nursing education. A wide range of countries was identified; these were categorized into developing country (0) and developed country (1) according to the World Economic Situation and Prospects 2015 Country Classification System for Developed and Developing Economies [41]. If a country was classified as an economy in transition, it was assigned to the developing country category.

Year of immigration was measured as a continuous variable by asking the participants to write the year they immigrated to Canada. The responses were collapsed into two categories: 1 = <2002 and 2 = ≥2002 to reflect the date of the IRPA in Canada.

Regulatory status was represented with three categories. Participants indicated the type of permanent nursing license they held in Canada at the time of the survey: RN, LPN, or RPN.

Categories for jurisdiction of registration and employment reflected the 10 provinces and 3 territories of Canada. Since it is required to hold a license to practice nursing in the jurisdiction in which the nurse works, the participants were asked to indicate where they were employed in: Alberta, British Columbia, Manitoba, New Brunswick, Newfoundland and Labrador, Nova Scotia, Ontario, Prince Edward Island, Quebec, Saskatchewan, or the territories including Nunavut, the Northwest Territories, and the Yukon. The numbers of participants who indicated they were employed in New Brunswick, Newfoundland and Labrador, Nova Scotia, or Prince Edward Island were small; thus, we collapsed these responses into the category Atlantic Provinces. No participants indicated their primary jurisdiction of employment was the Northwest Territories, Nunavut, or the Yukon.

#### Human capital variables

Nursing education, professional experience, and language proficiency were used to measure the human capital characteristics of IENs. Nursing education was assessed with three categories: diploma (nonuniversity degree), baccalaureate degree, and masters or PhD. Responses were collapsed into the following: diploma, nonuniversity degree (0) and university degree including baccalaureate degree, masters, or PhD (1). Professional experience was measured as the number of years and months of professional experience they had at the time of immigration. The responses were categorized into <3 years, 3–5 years, and >5 years [42]. Language proficiency in the two official languages of Canada was operationalized as the level of knowledge the participants had with each language (English and French). Participants indicated the type of experience they had with each language, separately, before immigrating to Canada: (1) English language proficiency included the following categories: 1 = no knowledge at all, 2 = minimal knowledge, 3 = second language, 4 = first language only, 5 = language of education only, and 6 = language of education and first language. The responses were collapsed into the following: 0 = no or limited knowledge (1 and 2) and 1 = moderate to highly knowledgeable (3–6). (2) The French language proficiency variable was measured and coded in the same manner.

#### Formal and informal assistance variables

Four variables operationalized participants’ receipt of formal (involvement in programs) or informal (help from individuals) assistance to prepare for the licensure exam or to find work as regulated nurses in Canada: bridging program participation, Canadian nursing experience, help studying for the licensure exam, and help finding their first job as regulated nurses in Canada. Bridging program participation was measured as a dichotomous variable (0 = no, I did not participate, and 1 = yes, I participated). Canadian nursing experience was measured as a dichotomous variable (0 = no, I did not have, and 1 = yes, I had experience) prior to writing and passing the licensure exam. Help studying for the licensure exam was measured by whether or not participants had help studying or preparing for the nursing licensure exam (0 = no and 1 = yes). Help finding their first job was measured by asking the participants if they had help (0 = no, I did not have help, and 1 = yes, I had help) from family, friends, or colleagues living in Canada to find their first job as a regulated nurse in Canada.

#### Dependent variables

Two dependent variables were used to measure IENs’ workforce integration, defined as acquiring professional recertification and securing employment as regulated nurses in Canada. Acquiring professional recertification was operationalized as successful results on the licensure exam on the first attempt, measured as a dichotomous variable (0 = no, I did not pass on the first attempt, and 1 = yes, I passed on the first attempt). Securing employment as regulated nurses in Canada was operationalized as the level of difficulty IENs experienced in securing their first job. It was assessed by soliciting IENs’ perceptions of their level of difficulty by using a numeric rating scale anchored with 0 = easy and 10 = very difficult to secure their first job. The responses were collapsed into the following: 0 = easy (0–4) and 1 = difficult (5–10).

### Data analysis

Data were analyzed using SPSS 24. Descriptive statistics, including frequencies and measures of central tendency (mean) and dispersion (SD), were computed to characterize the demographic and human capital profile of IENs and their responses to the survey items. Since the IEN population and the types of assistance available to them may have changed since the institution of the IRPA in 2002, cross tabulations and independent sample *t* tests were used to compare the responses of these two groups of IENs (i.e., those who immigrated before and after 2002).

Logistic regression analyses were applied to identify significant predictors of the dichotomous dependent variables of professional recertification and employment. The independent variables were informed by the literature and included the human capital characteristics of nursing education, professional experience, language proficiency (English and French), and forms of assistance including bridging program participation, Canadian nursing experience, and help studying for the licensure exam or help finding their first job as a regulated nurse in Canada.

To prepare for logistic regression modeling and rule out multicollinearity among the independent variables, the association among independent variables or predictors, which were categorical, was examined using chi-square analyses. Only the independent variables that were statistically uncorrelated were entered for model testing [43]. The set of independent variables differed for the two dependent variables. For passed the licensure exam on the first attempt, professional experience and help studying for the licensure exam were statistically uncorrelated and entered as predictors. For difficulty securing their first job as a regulated nurse, the independent variables were professional experience, bridging program participation, and help finding their first job, which were statistically uncorrelated and added to the regression model. For both regression models, year of immigration was entered in the first block (to control for its influence) while the other predictors were entered simultaneously (using forced entry) in the second block (to examine their contribution above and beyond the year of immigration).

### Ethical consideration

Approval to conduct the study was obtained from three research ethics boards: L’Université du Québec en Outaouais (CER 1767), the University of Ottawa (J08-12-16B), and l’Université de Montréal (13-119-CERES-R). Return or completion of the questionnaire indicates implied consent. All questionnaires (electronic and paper) were anonymous; no names were associated with the data. Participant ID numbers were assigned automatically when the IENs completed the questionnaire.

## Results

### Response rate

More than one quarter (*N* = 3794; 28%) of the 13 748 IENs approached responded to our invitation to take part in the study; however, 920 (24%) IENs were excluded for different reasons. A few (*n* = 87, 2%) IENs logged on to the survey website but did not respond to any questions. Sixty-nine (1%) logged on to the survey website but were not permitted to complete the survey online because they indicated they had already been contacted by another nursing regulatory body and participated. The remaining 764 (21%) did not meet the following eligibility criteria: 217 (6% of 764) did not have a permanent license to practice nursing in Canada, 223 (6%) were not employed as a regulated nurse at the time of the survey, and 324 (9%) were not immigrants to Canada.

Of the remaining 2874 IENs, 2244 (78%) responded to all the survey questions and 630 cases had incomplete responses. Of the cases with incomplete responses, 6% (*n* = 181) were eliminated from the sample for having had greater than 50% missing responses. No clear pattern could be identified that explained why these participants elected not to answer more than half of the survey questions.

**Fig. 1:**
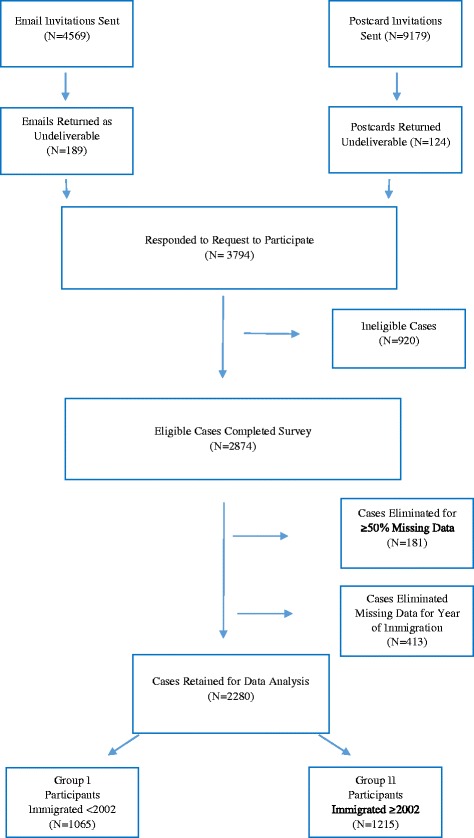
Sampling frame

For 15% (*n* = 413) of the remaining 2693 cases, the participants did not provide their date of immigration, a variable of interest in this study, making it difficult to classify them into the group of those who immigrated before or after 2002. Therefore, these cases were eliminated. As a result, the study sample consisted of 2280 IENs (80%, of 2874 who completed the survey). Figure 1 provides a flow diagram depicting the sampling strategy for this study.

### Demographic characteristics

**Fig. 2:**
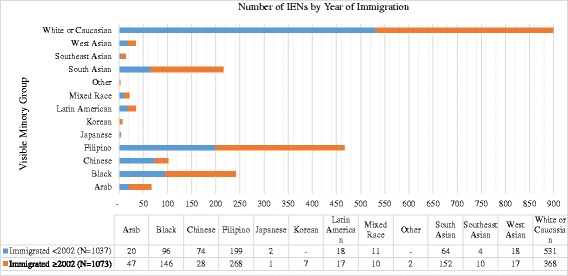
Numbers of IENs per visible minority group by year of immigration (*N* = 2210)

Table 1 summarizes the descriptive statistics for all variables, presented for the total sample and the group of IENs who immigrated prior to and after 2002. The participants were mostly middle-aged (*M* = 47 years, *SD* = 11), female (*n* = 1943, 85.2%), and self-identified as a visible minority (*n* = 1311, 57.5%). About half of the IENs received their basic nursing education in a developing country, mostly the Philippines and India (*n* = 1241, 54.4%). As Fig. 2 demonstrates, Filipinos represent the largest visible minority group, followed by Blacks and South Asians.

**Table 1:** Study variables for the sample and by year of immigration

Variable	Sample (*N* = 2280)	Immigrated <2002 (*N* = 1065)	Immigrated ≥2002 (*N* = 1215)	*a*
Demographic characteristics				
Age, mean (SD)	47.36 (11)	54.5 (8.81)	41 (8.65)	*t* = 35.79***
Gender, *n* (%)				*χ* ^2^ = 73.6***
Female	1943 (85.2)	976 (91.6)	967 (79.6)	
Male	280 (12.3)	64 (6.0)	216 (18.1)	
Visible minority, *n* (%)				*χ* ^2^ = 88.37***
Yes	1311 (57.5)	508 (47.7)	803 (66.1)	
No (White)	899 (39.4)	531 (49.9)	368 (30.3)	
Country of basic nursing education, *n* (%)				*χ* ^2^ = 97.83***
Developing country	1287 (56.4)	491 (46.1)	796 (65.5)	
The Philippines	556 (24.4)	198 (18.6)	358 (29.5)	
India	181 (7.5)	38 (3.6)	133 (10.9)	
China/Hong Kong	84 (3.7)	58 (5.4)	26 (2.1)	
Lebanon	42 (1.8)	14 (1.3)	133 (10.9)	
Jamaica	41 (1.8)	17 (1.4)	24 (2.3)	
Other developing countries	383 (16.8)	166 (15.5)	122 (10)	
Developed country	888 (38.9)	530 (49.8)	358 (29.5)	
United Kingdom	374 (16.4)	263 (29.5)	111 (9.1)	
France	131 (5.7)	32 (3)	99 (8.1)	
United States	107 (4.7)	61 (5.7)	46 (3.8)	
Poland	63 (2.8)	59 (5.5)	4 (<1)	
Australia	44 (1.9)	22 (2.1)	22 (1.8)	
Other developed countries	17 (1.4)	93 (<1)	76 (6.2)	
Regulatory status, *n* (%)				*χ* ^2^ = 76.13***
Registered nurse	2033 (89.2)	1013 (95.1)	1020 (84)	
Licensed practical nurse	220 (9.6)	43 (4)	177 (14.6)	
Registered psychiatric nurse	24 (1.1)	7 (<1)	17 (1.4)	
Province of registration and employment, *n* (%)				*χ* ^2^ = 294.55***
Alberta	471 (20.7)	192 (18)	279 (23)	
Atlantic Provinces	120 (5.3)	37 (3.5)	83 (6.8)	
British Columbia	305 (13.4)	146 (13.7)	159 (13.1)	
Manitoba	195 (8.6)	55 (5.2)	140 (11.5)	
Ontario	752 (33)	523 (49.1)	229 (18.8)	
Quebec	350 (15.4)	101 (9.5)	249 (20.5)	
Saskatchewan	80 (3.5)	7 (<1)	73 (6)	
Human capital characteristics				
Nursing education, *n* (%)				*χ* ^2^ = 240.78***
Diploma, nonuniversity	1087(47.7)	692 (65)	395 (32.5)	
Baccalaureate or higher	1172(51.4)	364 (34.2)	808 (66.5)	
Professional experience, *n* (%)				*χ* ^2^ = 29.51***
<3 years	567 (14.9)	320 (30.0)	247 (20.3)	
3–5 years	310 (13.6)	140 (13.1)	170 (14)	
>5 years	1386 (60.8)	596 (56.0)	790 (65)	
English language proficiency, *n* (%)				*χ* ^2^ = 5.42*
No or limited knowledge	459 (20.1)	239 (22.4)	220 (18.1)	
Moderate to highly knowledgeable	1721 (75.5)	791 (74.3)	930 (76.5)	
French language proficiency, *n* (%)				*χ* ^2^ = 37.36***
No or limited knowledge	1077 (47.2)	541 (50.8)	536 (44.1)	
Moderate to highly knowledgeable	326 (14.3)	101 (9.5)	225 (18.5)	
Formal and informal assistance				
Bridging program participation, *n* (%)				*χ* ^2^ = 37.85***
Yes	626 (27.5)	227 (21.3)	399 (32.8)	
No	1654 (72.5)	838 (78.7)	816 (67.2)	
Professional work experience in Canada, *n* (%)				*χ* ^2^ *= 0.729*, *p = * *ns*
Yes	772 (33.9)	351 (33)	421 (34.7)	
No	1274 (55.9)	604 (56.7)	670 (55.1)	
Help studying for nursing exam, *n* (%)				*χ* ^2^ = 12.45***
Yes	970 (42.5)	413 (38.8)	557 (45.8)	
No	1076 (47.2)	542 (50.9)	534 (44)	
Help finding the first job, *n* (%)				*χ* ^2^ = 14.84***
Yes	458 (20.1)	207 (19.4)	251 (20.7)	
No	1093 (47.9)	611 (57.4)	482 (39.7)	
Workforce integration				
Passed exam on the first attempt, *n* (%)				*χ* ^2^ = 14.63**
Yes	1499 (65.7)	661 (62.1)	838 (69)	
No	544 (23.9)	291 (27.3)	253 (20.8)	
Difficulty securing the first job, *n* (%)				*χ* ^2^ = 19.68**
Easy	596 (26.1)	242 (22.7)	354 (29.1)	
Difficult	1217 (53.4)	629 (59.1)	588 (48.4)	

*a* Based on *χ* ^2^ for categorical variables and independent *t* tests for continuous variables

*p < 0.05; **p < 0.01; ***p < 0.001;* ns*=nonsignificant

**Fig. 3:**
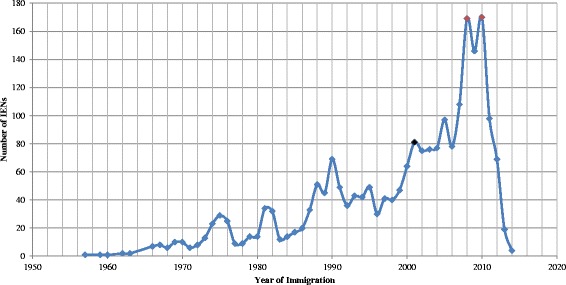
Number of IENs by year of immigration (*N* = 2280, *M* = 1999, *SD* = 11.45)

Most respondents were RNs (*n* = 2033, 89.2%), and the remaining were LPNs (*n* = 220, 9.6%) or RPNs (*n* = 24, 1.1%). One third of the sample reported their jurisdiction of registration and employment as Ontario (*n* = 752, 33%), followed by Alberta (*n* = 471, 20.7%), Quebec (*n* = 350, 15.4%), and British Columbia (*n* = 305, 13.4%). As Fig. 3 shows, the peak years for immigration were 2008 and 2010 (*M* = 1999, *SD* = 11.45).

Statistically significant differences in the demographic characteristics were detected between the two groups of participants. A higher proportion of the participants who immigrated ≥2002 was younger (*t* = 35.79, *p* < .001), was male (*χ* ^2^ = 73.6, *p* < .001), was from a visible minority group (*χ* ^2^ = 88.37, *p* < .001), and received their basic nursing education in a developing country (*χ* ^2^ = 100.98, *p* < .001).

The number of participants who were RNs was comparable between the two groups; however, greater numbers of participants who immigrated ≥2002 reported having a LPN license or a RPN license (*χ* ^2^ = 76.11, *p* < .001). Statistically significant differences were also noted in the jurisdiction of registration and employment with lower numbers of participants who immigrated ≥2002 in Ontario and higher numbers in other jurisdictions (*χ* ^2^ = 294.55, *p* < .001).

### Human capital characteristics

Upon immigration, slightly more IENs in the study sample had baccalaureate degrees, masters, or PhDs (*n* = 1172, 51.4%) and the majority had >5 years of professional experience (*n* = 1368, 60.8%). Three quarters (*n* = 1721) reported moderate or high proficiency in the English language. Far less reported being moderately or highly proficient in the French language (*n* = 326, 14.3%).

Nursing education and experience representing human capital characteristics differed significantly between the two groups. Compared to those who immigrated before 2002, more IENs who immigrated ≥2002 arrived in Canada with university (baccalaureate or higher) degrees in nursing (*χ* ^2^ = 240.78, *p* < .001) and with five or more years of professional experience (*χ* ^2^ = 29.51, *p* < .001). In addition, a greater proportion of immigrants ≥2002 were moderately or highly proficient in the English language as opposed to those who immigrated prior to 2002 (*χ* ^2^ = 5.42, *p* < .05). The proportion of participants who were moderately or highly proficient in the French language was also greater for the group who immigrated ≥2002 (*χ* ^2^ = 37.36, *p* < .001).

### Formal and informal assistance

Nearly three quarters (*n* = 1654, 72.5%) of the participants reported they did not take part in bridging programs. One third (*n* = 772, 33.9%) had Canadian nursing experience. Less than half (*n* = 970, 42.5%) received help studying for the licensure exam and had help with their job search (*n* = 458, 20.1%).

Statistically significant differences were noted between the two groups on receipt of formal or informal assistance to integrate into the workforce. Significantly higher numbers of participants who immigrated ≥2002 participated in bridging programs (*χ* ^2^ = 37.85, *p* < .001), had help studying for the licensure exam (*χ* ^2^ = 12.45, *p* < .001), and received assistance to find their first job as a regulated nurse (*χ* ^2^ = 14.84, *p* < .001).

### Workforce integration

Most (*n* = 1499, 65.7%) participants passed the licensure exam on their first attempt. Approximately one half of the participants (*n* = 1217, 53.4%) reported experiencing difficulty securing their first job as regulated nurses. However, a greater proportion of participants who immigrated ≥2002 passed the exam on their first attempt (*χ* ^2^ = 14.63, *p* < .01). This same group also reported less difficulty securing their first job as regulated nurses as opposed to the participants who immigrated earlier (*χ* ^2^ = 19.68, *p* < .01).

### Predictors of passing the licensure exam on the first attempt

Table 2 presents the human capital characteristic (professional experience) and the form of assistance (help study for the exam) found to be associated with passing the licensure exam on the first attempt. Since these factors varied significantly between the two groups of participants, the influence of the year of immigration was controlled for by entering this variable in the first block. The predictors, professional experience and help studying for the exam, were entered simultaneously in the second block. The results indicate that having 3–5 years of professional experience (OR = 1.612, CI [1.252, 2.075]) and help studying for the licensure exam (OR = 2.373, CI [1.929, 2.918]; both *p*’s < .001) significantly predicted passing the exam on the first attempt. The model (Nagelkerke *R* ^2^) was .068 and correctly classified 73.7% of the cases.

**Table 2:** Logistic regression model for passed licensure exam on the first attempt (*N* = 2020)

Predictor	95% CI for odds ratio			
	*β*(SE)	Lower	Odds ratio	Upper
Professional experience: 3–5 years	.477(.129)***	1.252	1.612	2.075
Professional experience: >5 years	.282(.163)	0.963	1.326	1.826
Help studying for the exam: yes	.864(.106)***	1.929	2.373	2.918
Constant	−.297			
*χ* ^2^ (df)	96.132(4)***			
Nagelkerke *R* ^2^	.068			
% of respondents who passed the registered nurse licensure examination on the first attempt	73.7%			

Control variable (not shown in the table): year of immigration coded as 0 = <2002 and 1 = ≥2002. Previous nursing experience coded as 1 = <3 years (reference variable), 2 = 3–5 years, and 3 = >5 years; help studying for the exam coded as 0 = no (reference variable) and 1 = yes

*CI* confidence interval, *β* beta coefficient, *SE* standard error, *χ* ^*2*^ chi-square, *df* degrees of freedom

***p < .001

### Predictors of difficulty securing their first job

The human capital characteristic (professional experience) and forms of assistance (bridging program participation and help finding their first job) associated with difficulty securing their first job are presented in Table 3. After controlling for year of immigration, bridging program participation (OR = 0.583, CI [0.452, 0.752] and help finding their first job (OR = 1.919, CI [1.507, 2.442]; both *p*’s < .001) significantly predicted difficulty securing their first job as a regulated nurse in Canada. Professional experience was not a significant predictor of IENs securing employment. The model (Nagelkerke *R* ^2^) was .064 and correctly classified 65.5% of the cases.

**Table 3:** Logistic regression model for difficulty securing the first job as a regulated nurse in Canada (*N* = 1351)

	95% CI for odds ratio			
Predictor	*β*(SE)	Lower	Odds ratio	Upper
Professional experience: 3–5 years	.075(.137)	0.824	1.078	1.409
Professional experience: >5 years	−.063(.180)	0.660	0.939	1.334
Bridging program participation: yes	−.540(.130)***	0.452	0.583	0.752
Help finding the first job: yes	.652(.123)***	1.507	1.919	2.442
Constant	.226			
*χ* ^2^ (df)	65.187***			
Nagelkerke *R* ^2^	.064			
% of respondents who had difficulty finding their first job	65.5%			

Control variable (not shown in table): year of immigration coded as 0 = <2002 and 1 = ≥2002. Previous nursing experience coded as 1 = <3 years (reference variable), 2 = 3–5 years, and 3= >5 years; bridging program participation coded as 0 = no and 1 = yes; help finding the first job coded as 0 = no and 1 = yes

*CI* confidence interval, *β* beta coefficient, *SE* standard error, *χ* ^*2*^ chi-square, *df* degrees of freedom

***p < .001

## Discussion

Using data from the first pan-Canadian cross-sectional study of IENs, this study sought to provide a profile of the demographic and human capital characteristics of Canadian IENs and to explore recent changes to the profile. Predictors of IENs’ professional recertification and employment in Canada were identified.

### Response rate

More than one quarter (28%) of the IENs we contacted responded to our request to participate in the study. Although the accrued sample reflects our estimated sample size and provides sufficient power for the statistical tests used in this study, the response rate was slightly below that of other studies that surveyed the same nursing occupational groups in Canada [44]. This may be due to the methods used to identify and approach IENs, which varied across jurisdictions [38].

### Demographic profile

The results reveal the Canadian IEN workforce is largely female, middle-aged, and racially diverse. Although males represent a higher proportion of those who immigrated ≥2002, the IEN population remains female dominated. The Philippines educated the largest proportion of IENs, making up approximately one fifth of the Canadian supply. A shift in the country of education is notable, in that the other primary source countries for IENs are no longer westernized developed countries such as the United Kingdom or the United States but rather a variety of developing countries throughout Africa, Asia, Europe, and the Middle East.

The change in country of education is most likely the results of global migration trends with migrants moving from developing to developed economies in search of well-paying jobs, professional development or career advancement opportunities, improved quality of life, and stable socio-political environments [45]. The rising numbers of Filipino IENs most likely reflect the international recruitment initiatives, some private and others spearheaded by provincial health agencies in Canada, as well as the Philippines’ approach to produce nurses for global export. The Philippines view nurses who work abroad as potential sources of remittance income, an important contributor to the nation’s economy [46].

Most IENs in our study became RNs in Canada. Trends were noted in the data for greater numbers of recent arrivals to obtain LPN and RPN licenses. Little is known about the reasons IENs choose to become LPNs or RPNs, which could be personal, professional, financial, or social. Interestingly, this finding may signify the existence of a credentialing pathway where IENs become LPNs on their way to professionally recertifying as RNs, an area that requires further investigation [47].

The jurisdictions with the highest numbers of IENs in our study were Ontario, Alberta, and Quebec, a finding that is supported by CIHI data [34]. Interestingly, the number of participants from Quebec was higher for the ≥2002 group. This could reflect the province’s approach to immigration and regulatory body’s agreement with France permitting the active recruitment of French nurses to work in the Quebec healthcare system as well as the effectiveness of educational programs and employer’s supports that accompany the agreement [48]. In recent years, many IENs in Quebec have benefited from pre-immigration job placement, mandatory bridging program participation through provincial colleges, probationary licenses, and employer-sponsored orientation programs [35]. Thus, the higher number of participants from Quebec may reflect the success of the accompanied programs in providing IENs with the opportunity to upgrade their language proficiency and nursing competencies to Canadian standards and to acquire Canadian nursing experience prior to taking the licensure exam. Both types of assistance are thought to facilitate IENs’ professional recertification and employment.

### Human capital profile

Overall, recently arrived IENs are highly educated, professionally experienced, and proficient in the English and/or French language. These findings are consistent with the changes made to the immigrant selection process post IRPA with a greater number of points being allotted for the immigrants’ education, language ability, and professional experience [18]. The large proportion of IENs with baccalaureate degrees reflects the entry to practice requirements for RNs in most jurisdictions of Canada, especially since the majority of our respondents were RNs.

### Formal and informal assistance

Regardless of when they formally immigrated, the majority of IENs in our study did not participate in bridging programs or have Canadian nursing experience prior to passing the licensure exam. A small increase in the proportion of IENs who participated in bridging programs prior to passing the licensure exam was evident, especially for the group who migrated ≥2002. Those who had Canadian nursing experience prior to passing the licensure exam remained relatively stable over time. There are several reasons for the overall lower participation in bridging programs including the limited availability and regional maldistribution of bridging programs with most being situated in urban centers [49], the length of time required to complete a program, and the associated loss of income and tuition costs [50]. Since not all bridging programs include clinical placement, IENs may have limited opportunities to acquire Canadian nursing experience prior to professionally recertifying. The slight increase in bridging program participation evident in those who migrated ≥2002 could be the result of an increase in the number of available programs [28], many provided through funding from governmental programs such as the IEHP Initiative as well as Quebec’s requirement of IENs’ successful completion of a bridging program as a condition for acquiring professional recertification [51].

Informal forms of assistance, such as having help from their social network of friends and colleagues in Canada, are strategies that some but not all IENs use to pass the licensure exam and secure employment. These strategies may be most helpful to IENs who are unfamiliar with the exam content and testing methods used for the licensure exams and the processes used to search for employment in Canada. This finding is confirmed by previous qualitative research that theorized social capital as an important facilitator of IENs’ integration into the nursing workforce [32]. It also supports the higher weights post IRPA given to immigrants who have social networks in Canada [19].

### Predictors of professional recertification

Professional experience and help studying for the exam were significant predictors of IENs passing the licensure exam on their first attempt. IENs were 1.6 times more likely to pass the licensure exam on their first attempt if they had 3–5 years of professional experience before immigrating to Canada. IENs were 2.4 times more likely to pass the licensure exam on their first attempt if they had help studying for the exam.

These findings indicate the combination of a modest amount of professional experience and having help studying are important factors that support IENs’ success on the licensure exam. A modest amount of professional experience most likely reflects IENs who are relatively recent graduates. These IENs most likely have a current knowledge base consistent with the exam content and honed test taking skills, both factors believed to lead to exam success. Mentorship for exam preparation familiarizes IENs with how the questions are structured, response item format, Canadian-specific content, and occupational-specific language used in the licensure exam. Providing IENs with formal or informal assistance to update their knowledge in the form of study groups, review courses, and opportunities to receive coaching to refine their test taking skills are some strategies that may help more IENs pass the exam on the first attempt. Encouraging IENs to become familiar with the exam content, structure, and administration process and to solicit the mentorship from other IENs or licensed nurses while preparing for the exam are also recommended strategies that could lead to exam success. These strategies would be especially important for IENs who have been practicing longer or received their basic nursing education sometime before immigrating.

### Predictors securing employment

Bridging program participation and help from friends and colleagues in Canada were significant predictors if IENs had difficulty finding their first job as a regulated nurse in Canada. If IENs attended bridging programs, they were 0.58 times less likely to have difficulty finding their first job. However, those who had help from friends and colleagues in Canada were 1.9 more likely to have experienced difficulty. This suggests when IENs struggle to secure employment, they seek assistance from their social network in Canada to help them navigate their job search and the hiring process [32]. Bridging program participation may provide assurances to employers that IENs are prepared to work in Canada [9]. Both bridging program participation and having social networks may connect IENs with potential employers [33, 52].

### Implications for IEN workforce integration

The findings from this study signify that human capital characteristics are important facilitators of IENs’ workforce integration. Since most participants in our study were prepared at the baccalaureate level or higher, and had moderate to high levels of language proficiency, we are unable to determine the extent to which these attributes influence IENs’ professional recertification and employment. We do however suspect that the educational preparation and language proficiency of our participants did play a role in their success on the licensure exam. This assumption would be consistent with previous research [24]; however, additional studies are needed to clarify the level of language proficiency required to pass the licensure exam and methods to determine its influence on IENs’ capability to provide safe patient care.

We did determine that 3–5 years of professional experience upon immigration was influential to IENs’ passing the licensure exam on the first attempt. This is because these participants had the combination of relatively recent educational preparation, making them uniquely situated to achieve success on the exam. This conclusion is supported by findings from research conducted in Quebec that revealed IENs with numerous years of professional experience prior to immigrating had greater difficulty acquiring nursing skills in Canada and adjusting to the way nursing was practiced in the province’s healthcare settings [53]. The findings are also aligned with the literature about nurses’ acquisition of clinical competency, indicating that nurses with 3–5 years of professional experience are proficient clinicians and have a sufficient amount of experience to formulate clinical decisions [42], factors that may have contributed to their success on the licensure exam [1]. Therefore, it could be surmised that relatively recent university graduates, with moderate to high language proficiency and professional experience, are ideal candidates for international migration and employment. However, further research is required to substantiate this assumption.

Some IENs benefited from educational opportunities such as bridging programs to upgrade their professional competencies to the country’s standards. However, participation in bridging programs was not significantly associated with passing the licensure exam on the first attempt, meaning that many IENs did not require this type of support. This finding suggests that bridging program participation is influential for IENs whose educational and/or learning needs are great or are struggling. Further research is needed to identify promising bridging program practices and how they facilitate IEN workforce integration. Clarifying the expected outcomes associated with IEN bridging program participation is also needed.

Bridging program participation did play a significant role in making the job search for IENs easier. This finding points to the importance of IENs having “social networks” to help them navigate their job search. When IENs do not have social networks, providing forums (in-person or virtual) and opportunities for IENs to develop their social capital are recommended. CARE Centre for Internationally Educated Nurses, in Ontario [54], is an example of a current Canadian initiative for IENs to cultivate their professional and social relationships and to obtain the information needed to navigate their job search and integrate into the regulated nursing workforce. Other countries could consider developing similar programs to support the integration of IENs who migrate to their countries.

Our findings demonstrate that IENs are not homogenous in that they are educated in and migrate from many different countries with varying types of education and healthcare systems throughout the world. A “one size fits all” approach may not be appropriate for meeting the needs of all IENs [4]; rather, consideration could be given to adopting a case management approach to assisting IENs with achieving workforce integration [55]. Selecting and tailoring education and the form of assistance to meet the individualized needs of IENs may prove to be a more “fruitful” method for facilitating their workforce integration [35]. Since bridging programs are mostly located in major urban centers, they are often inaccessible to IENs who live in rural or remote areas of the country. Using technology to reach IENs who have settled outside of urban centers is one potential strategy that may help prevent IENs in these areas from dropping out of the profession and ending up in survival jobs. This same strategy could be used for IENs who have not yet immigrated, as a method of helping to identify areas for remedial attention, thus facilitating their timely entrance into the destination countries’ nursing workforces.

### Strengths and limitations

The major strength of this study is that it generates evidence about a large and relatively understudied health provider group in Canada. The findings from this first cross-sectional survey about IENs who have formally immigrated to Canada provide a profile of the demographic and human capital characteristics of the population and discuss how the characteristics of the profile have changed in recent years. Another strength is the questionnaire used in this study, in that it was carefully constructed and its content was validated with a panel of experts.

The limitations of this study include a potential sample selection bias. Participants represent those who agreed to be contacted for research purposes and who were successful in obtaining licenses and employment as regulated nurses at the time of the study, thus eliminating those who are unemployed or are employed with temporary work visas. Methodologically, most predictors were assessed with one item, which makes it difficult to determine the extent of measurement error [56]. The multiple tests for the comparison between the two groups could have led to type 1 error in that some of the differences could be due solely to chance [57].

## Conclusions

Professional recertification and securing their first jobs as regulated nurses are essential steps towards leveraging IENs’ human capital and retaining IENs in the destination country. This study found that many IENs became professionally recertified and secured employment easily while others required informal and formal assistance to integrate into the regulated nursing workforce. Other countries can use this information when examining the needs of their IENs and for developing strategies to help them integrate into their health workforces.

When IENs can practice their profession in their new countries, the healthcare systems profit by gaining valuable health human resources who can provide culturally sensitive patient care [58]. IENs as new immigrants also benefit by earning the financial resources needed to care for their families and to contribute economically to their new communities and country [59].

## Abbreviations

IENs: Internationally educated nurses
IRPA: Immigration and Refugee Protection Act
LPN: Licensed practical nurse
RN: Registered nurse
RPN: Registered psychiatric nurse

## Footnotes

1 Since 2014, the National Nursing Assessment Service coordinates a consistent national approach for IENs seeking registration/licensure to practice in Canadian jurisdictions.

2 In Quebec, successful demonstration of proficiency in the French language is required to obtain a permanent license to practice nursing in the province.
